# How to play the final chess match—or at least lose with dignity

**DOI:** 10.1080/17453674.2021.1959159

**Published:** 2021-07-28

**Authors:** Cecilia Rogmark, Niels Lynøe

**Affiliations:** a Department of Orthopaedics, Lund University, Skane University Hospital, Malmö, Sweden;; b Department of Learning, Informatics, Management and Ethics, Stockholm Centre for Healthcare Ethics, Karolinska Institutet, Stockholm, Sweden

**Figure:**
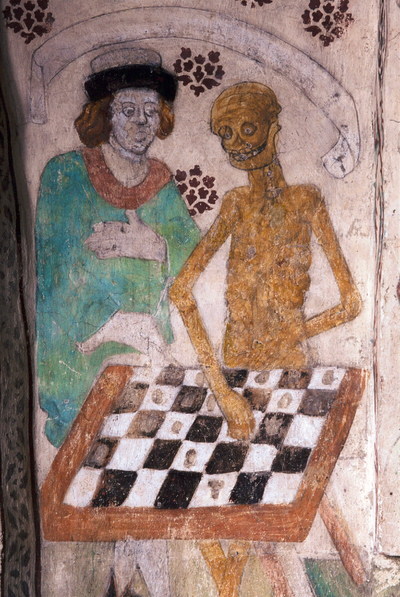

Death playing chess. Mural painting by Albertus Pictor around 1480, Tдby Church, Sweden

In this issue of *Acta Orthopaedica*, Wijnen et al. (2021) report that after nonoperative treatment of hip fractures nine-tenths died during the first month although one-third lived independently before their fracture. This may be startling, but we cannot evade the topic, as we are experiencing a rapid increase of very old citizens who bring their generation’s way of thinking into the discussion of end-of-life care.

An old person, a physician, an acute fracture, and death: a serious plot, leading to very different stories in different countries. Sometimes, other actors set the scene: legislation, tradition, family members. The rate of nonoperative treatment of hip fracture varies from 1% to 25% (SFR 2019) (Amrayev et al. 2018, Tomioka et al. 2020), with lowest rates in northern Europe, where non-surgery is regarded as a feature from the past (Jensen and Tøndevold 1980), even if the pandemics have challenged this picture (Mi et al. 2020). Therefore, the increasing interest in the Netherlands in nonoperative treatment is conspicuous (van de Ree et al. 2017, Joosse et al. 2019, van der Zwaard et al. 2020).

How well does shared decision-making work in an acute setting? The elderly patient with a hip fracture is in an acute crisis, perhaps ready to choose death over the assumed consequences of a hip fracture (Salkeld et al. 2000). Being in pain in a stressful emergency room, she fears she will lose her independence. She might be so modest that she does not want to be a nuisance to the healthcare system or her family. Laypersons, i.e., family, will be more prone to see risks than benefit with acute surgery, wanting to spare their old family member such strain. Healthcare staff are supposed to respect competent patients’ right to participate in decision-making regarding treatment that concerns the patient—the autonomy principle (Gillon 2003). When in medical need, the patient should be offered treatment, but also has a right to decline treatment. This negative right of a competent patient should be respected.

But a surgeon who considers surgery as a way to reduce pain and improve mobility during the remaining lifespan of the patient will find such a decline frustrating. To avoid decision-making based on prejudices regarding bad outcome, the physician has to listen carefully to the patient’s ideas, worries, expectations, prejudices, and preferences—without interrupting or arguing with the patient. After summarizing and acknowledging the input from the patient—the first step in patient-centered care (Hedberg and Lynøe 2013)—the physician should inform the patient of what is correct and incorrect regarding factual aspects, avoiding tacitly impregnating her or his own values in this presentation (Lynøe et al. 2018). The physician must also ensure that the patient actually understood the information regarding the consequences of the treatment alternatives. If, after the patient-centered procedure, the patient still declines surgery we have to respect a competent patient’s right to do so, when based on an informed and shared decision.

When interpreting scientific results, we have to bear in mind basic differences between countries and their healthcare systems. Wijnen et al. (2021) report an extremely high mortality after non-surgical treatment. Even if 29% of these Dutch patients lived independently before their hip fracture, 87% died during the first month. Previous reports describe a 1-month mortality of 19–36% after nonoperative treatment (Hossain et al. 2009, Loggers et al. 2020), but the case-mix differs. The underlying philosophy of the current study seems to be that suffering a hip fracture can be taken as a possibility to shorten the life of an individual who “has no wish to prolong life.” In other countries, the endeavor is the contrary, for example as stated by Berry et al. (2018): “Surgical repair of a hip fracture was associated with lower mortality among nursing home residents with advanced dementia and should be considered together with the residents’ goals of care in management decisions.” It may be that our understanding of advance care planning differs more than we at first realize (van der Steen et al. 2016). Indeed, culture and legislation differ between counties, and the Netherlands has both leading research activity in advance care planning and a transparent practice of euthanasia (Onwuteaka-Philipsen et al. 2012, Rietjens et al. 2017).

In the future we will encounter more patients with hip fractures, most certainly individuals who will express other requests for their final stage of life, and not just suffer in silence. The orthopedic surgeon should prepare by understanding that the attitudes to advance care planning vary, that patients may prefer not to prolong life and ask for palliative care. Others may have an unrealistic view of what could be done to prolong life. Regardless of direction, applying a patient-centered approach, information procedure, and shared decision-making might decrease the orthopedic surgeon’s frustration.
